# Supplementary material for: Order–disorder phase transition and elastic-to-plastic vortex creep crossover in a triclinic iron pnictide superconductor (Ca0.85La0.15)10(Pt3As8)(Fe2As2)5
Source: Sci Rep. 2023 Sep 28;13:16273. doi: 10.1038/s41598-023-43424-z (PMC10539531; doi:10.1038/s41598-023-43424-z)
Supplement: Supplementary file 1 — Supplementary Figures. [file 41598_2023_43424_MOESM1_ESM.pdf]

Order-disorder phase transition and elastic-to-plastic vortex creep crossover in a triclinic iron pnictide superconductor $(Ca_{0.85}La_{0.15})_{10}Pt_3As_8Fe_2As_2)_5$

Shyam Sundar [1,2], P. V. Lopes [1], S. Salem-Sugui, Jr. [1], Z. -Z. Li [3], W. -S. Hong [3,4], H. -Q . Luo [3],S. -L. Li [3], and L. Ghivelder [1]

[1] Instituto de Fisica, Universidade Federal do Rio de Janeiro, 21941-972 Rio de Janeiro, RJ, Brazil
[2] School of Physics and Astronomy, University of St Andrews, St Andrews KY16 9SS, Scotland, United Kingdom.
[3] Beijing National Laboratory for Condensed Matter Physics, Institute of Physics, Chinese Academy of Sciences,
Beijing 100190, China
[4] International Center for Quantum Materials, School of Physics, Peking University, Beijing 100871, China

Supplementary Information

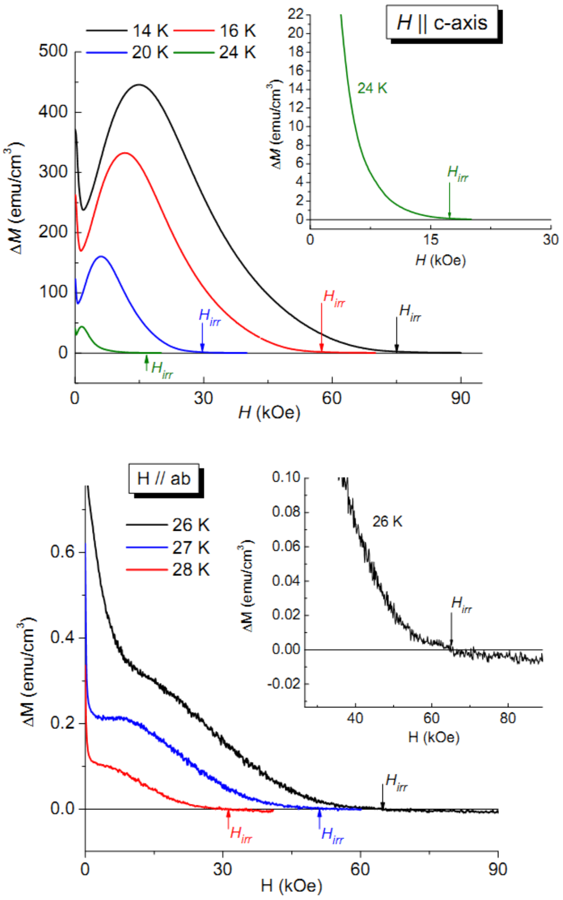

Figure S1: Magnetic field dependence of the difference between the field-increasing and field-decreasing branches of the isothermal magnetization curves, ΔM (H), at different temperatures. Arrows indicate the irreversibility field, where ΔM =0, for H||c (top panel), and for H||ab (bottom panel).

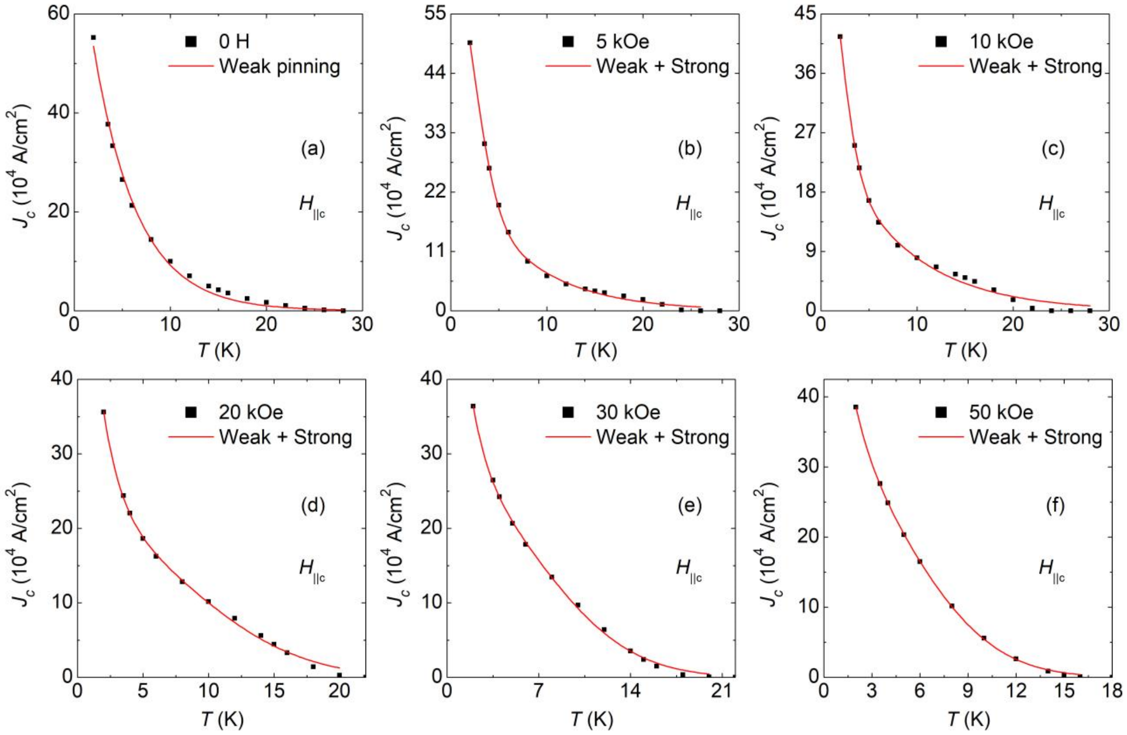

Figure S2: Temperature dependence of critical current density, $J_c(T)$, at different magnetic fields for $H \parallel$ c. In each panel, the solid line represent the fit of the data using models considering the weak, $J_c^{weak}(T) = J_c^{weak}(0)e^{-T/T_0}$, and strong pinning, $J_c^{str}(T) = J_c^{str}(0)e^{-3(T/T^*)^2}$. Here, $J_c^{weak}(0)$, and $J_c^{str}(0)$ are the values of J_c at zero temperature in respective expressions, and T_0 and T^* are the characteristic pinning energies for weak and strong pinning centers respectively.

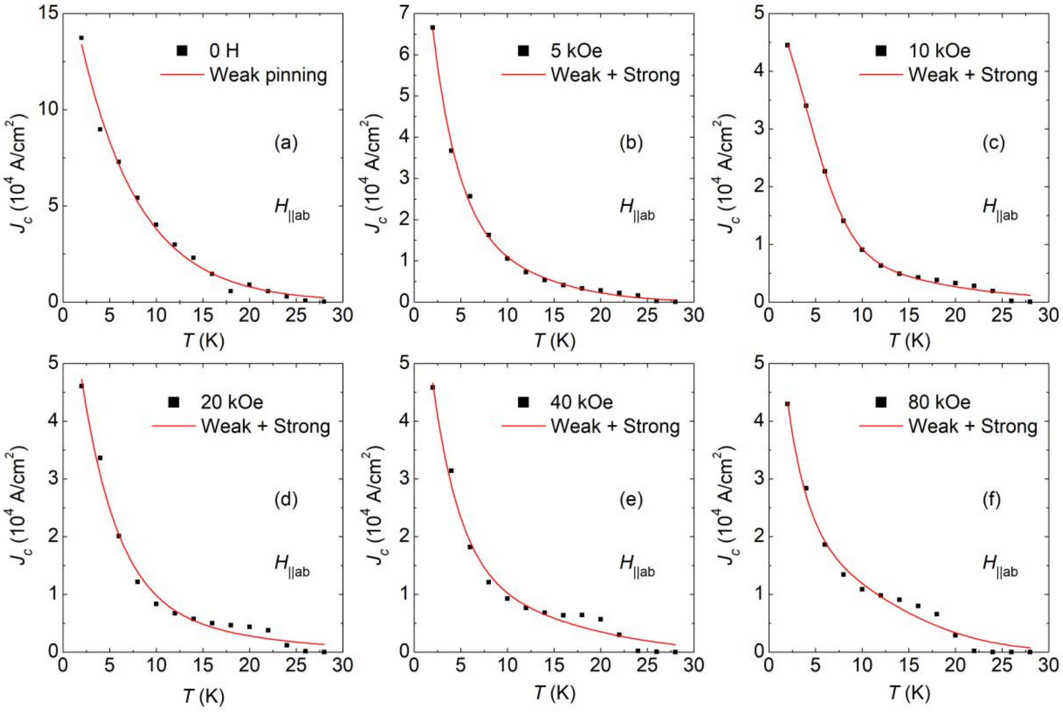

Figure S3: Temperature dependence of critical current density, $J_c(T)$, at different magnetic fields for $H \parallel$ ab. In each panel, the solid line represent a fit of the data using models considering weak, and strong pinning centers using expressions given in the caption of Fig. S2.
